# Socioeconomic Inequality in Long-Term Care: A Comparison of Three Time Periods in the Netherlands

**DOI:** 10.1093/geroni/igaa057.2569

**Published:** 2020-12-16

**Authors:** Jens Abbing, Bianca Suanet, Marjolein Broese van Groenou

**Affiliations:** 1 Vrije Universiteit, Amsterdam, Netherlands; 2 Vrije Universiteit Amsterdam, Amsterdam, Noord-Holland, Netherlands; 3 VU University, Amsterdam, Noord-Holland, Netherlands

## Abstract

This study aims at investigating to what extent inequalities in the use of formal, informal and privately paid care have changed over time. Data from the Longitudinal Aging Study Amsterdam (LASA) was used from three points in time (1995, 2005 and 2015) that capture distinct periods in the recent development of the Dutch long-term care system. In particular, the reforms of 2007 and 2015 might have impacted care uses. All participants (N = 1810) were living at home and between the age of 75 and 85 at measurement. The results indicate that, adjusted for health and partner status, formal, informal and privately paid care have decreased over time. Socioeconomic differences in informal care use have increased over time, but no change was found for privately paid or formal care use. These findings suggest that changes in the LTC system and long-term care resources in particular benefit lower socioeconomic groups.

